# Bovine Serum Albumin-Conjugated Ferrimagnetic Iron Oxide Nanoparticles to Enhance the Biocompatibility and Magnetic Hyperthermia Performance

**DOI:** 10.1007/s40820-015-0065-1

**Published:** 2015-10-15

**Authors:** Viveka Kalidasan, Xiao Li Liu, Tun Seng Herng, Yong Yang, Jun Ding

**Affiliations:** 1 Department of Materials Science and Engineering, Faculty of Engineering, National University of Singapore, 7 Engineering Drive 1, Singapore, 117574 Singapore; 2 Shaanxi Key Laboratory of Degradable Biomedical Materials, School of Chemical Engineering, Northwest University, Xi’an, 710069 Shaanxi People’s Republic of China

**Keywords:** Magnetic hyperthermia, Ferrimagnetic iron oxide nanoparticles, Bovine serum albumin, Haemolysis, Cell viability, Specific absorption rate

## Abstract

**Abstract:**

Magnetic hyperthermia is a fast emerging, non-invasive cancer treatment method which is used synergistically with the existing cancer therapeutics. We have attempted to address the current challenges in clinical magnetic hyperthermia-improved biocompatibility and enhanced heating characteristics, through a single combinatorial approach. Both superparamagnetic iron oxide nanoparticles (SPIONs) of size 10 nm and ferrimagnetic iron oxide nanoparticles (FIONs) of size 30 nm were synthesized by thermal decomposition method for comparison studies. Two different surface modifying agents, viz, Cetyl Trimethyl Ammonium Bromide and 3-Aminopropyltrimethoxysilane, were used to conjugate Bovine Serum Albumin (BSA) over the iron oxide nanoparticles via two different methods—surface charge adsorption and covalent amide bonding, respectively. The preliminary haemolysis and cell viability experiments show that BSA conjugation mitigates the haemolytic effect of the iron oxide nanoparticles on erythrocytes and is non-cytotoxic to the healthy Baby Hamster Kidney cells. It was observed from the results that due to better colloidal stability, the SAR value of the BSA-iron oxide nanoparticles is higher than the iron oxide nanoparticles without BSA, irrespective of the size of the iron oxide nanoparticles and method of conjugation. The BSA-FIONs seem to show improved biocompatibility, as the haemolytic index is less than 2 % and cell viability is up to 120 %, when normalized with the control. The SAR value of BSA-FIONs is 2300 W g^−1^ when compared to 1700 W g^−1^ of FIONs without BSA conjugation. Thus, we report here that BSA conjugation over FIONs (with a high saturation magnetization of 87 emu g^−1^) provide a single combinatorial approach to improve the biocompatibility and enhance the SAR value for magnetic hyperthermia, thus addressing both the current challenges of the same.

**Graphical Abstract:**

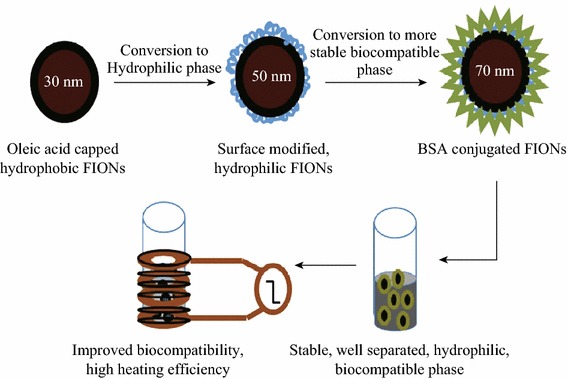

## Introduction

Cancer is a global killer disease. All the existing cancer treatment strategies like chemotherapy, radiotherapy, and hyperthermia techniques [1, 2] aim to alleviate cancer. Magnetic hyperthermia is a non-invasive technique which is better than the other hyperthermia techniques as it ensures targeted heating of the tumor tissue [3, 4]. Magnetic nanoparticles are a pre-requisite for an efficient magnetic hyperthermia system. Iron oxide nanoparticles with suitable surface modification and functionalization have a plethora of applications in magnetic resonance imaging (MRI), targeted drug delivery, cell separation, cell sorting, especially in magnetic hyperthermia [5–7], etc. In magnetic hyperthermia technique, iron oxide nanoparticles are injected into the tumor site and are subjected to an external alternating current (AC) magnetic field which raises the temperature of the tumor site up to 42–46 °C. This causes tumor cell death due to necrosis, incase of high dose of temperature–time [8–10] or apoptosis due to mild exposure and cell sensitization to chemotherapy and radiotherapy incase sublethal dose of temperature–time [11, 12]. Since iron oxide nanoparticles are injected into the biological system to bring about the temperature raise, a system with a very good biocompatibility and heating efficacy is the fundamental requirement for an efficient magnetic hyperthermia system. Thus, the current challenges to be addressed in the field of clinical magnetic hyperthermia are improved biocompatibility and enhanced heating characteristics.

Extensive research has been done to improve the biocompatibility and blood circulation of iron oxide nanoparticles by numerous surface modification and functionalization strategies for various biomedical applications [13–18]. Similarly, various attempts have been made to enhance the specific absorption rate (SAR) value of the iron oxide nanoparticles by controlling the aggregation, saturation magnetization, anisotropy, etc. [19–22]. The size and shape of the iron oxide nanoparticles can also be controlled in order to improve the SAR value and thereby enhance the heating characteristics [23]. Achieving both biocompatibility and improved heating efficacy without compromising on either factor poses a huge challenge in the research of clinical magnetic hyperthermia. This paper attempts to address both the pressing challenges by a single combinatorial approach.

Biocompatible iron oxide nanoparticles for magnetic hyperthermia using polymers like poly ethylene glycol (PEG), poly vinyl pyrolidone (PVP), poly ethyleneimine (PEI), biomacromolecules like proteins, aptamers, DNA, and surfactants like CTAB are well reported [24–26]. Combinatorial approach to simultaneously improve the biocompatibility and SAR value using noble metal like Platinum-coated iron oxide core–shell nanoparticles are also reported [27, 28]. Chemical surface modifying agents render good hydrophilicity and stability to the iron oxide nanoparticles. But they are cytotoxic when used beyond the optimum threshold level [29–31]. Therefore, biomacromolecules like DNA and proteins are preferred surface functionalization agents. For our work, we have chosen bovine serum albumin (BSA) as the biocompatibility agent. Albumin is a versatile protein which forms almost 55 % of blood plasma protein content and helps maintain the pH and osmotic pressure of blood [32, 33]. Thus, BSA conjugation improves the stealth characteristics of iron oxide nanoparticles and hence prolongs the blood circulation time [34, 35]. BSA is also reported to raise the temperature of a nanoparticle system under an applied AC magnetic field by the formation of isotropic clusters [36]. Previous reports of Human Serum Albumin (HSA)-conjugated superparamagnetic iron oxide nanoparticles (SPIONs) by Keshavarz et al. and BSA-conjugated SPIONs by Samanta et al. [37] also show that albumin conjugation improves the colloidal stability and thereby the SAR value for magnetic hyperthermia. In these reports, SPIONs were synthesized by inorganic co-precipitation method and albumin was conjugated by physical adsorption method. Thus, we have chosen BSA as our single preferred candidate for both improved biocompatibility and enhanced heating efficiency. Moreover ferrimagnetic iron oxide nanoparticles (FIONs) have higher saturation magnetization and hence better SAR value than SPIONs [38, 39]. The unique heat enhancement property of BSA adds further value to heating efficiency of FIONs. Therefore, BSA and FIONs are the candidates of interest for the single combinatorial approach to address the challenges in magnetic hyperthermia.

We have fabricated BSA-conjugated SPIONs of size 10 nm and FIONs of size 30 nm. The iron oxide nanoparticles were synthesized by thermal decomposition method using organic precursors [40, 41]. This is the highly preferred method for synthesizing iron oxide nanoparticles of uniform shape, size, and monodispersity when compared to the inorganic synthesis protocols [42–44]. Though inorganic synthesis protocols can generate hydrophilic iron oxide nanoparticles, the thermal decomposition method produces better quality hydrophobic iron oxide nanoparticles. The challenges in finding a suitable surface modifying agent to overcome the hydrophobicity of the as-synthesized iron oxide nanoparticles were met out. Two surface modifying agents, viz, Cetyl Trimethyl Ammonium Bromide (CTAB) and 3-aminopropyltrimethoxysilane (APTMS), were used to render preliminary hydrophilicity to the as-synthesized hydrophobic iron oxide nanoparticles and also to facilitate the conjugation of BSA by two different approaches—physical adsorption by surface charge interaction (CTAB-iron oxide nanoparticles) and strong covalent amide bonding by 1-ethyl-3-(-3-dimethylaminopropyl) carbodiimide hydrochloride (EDC) method (APTMS-iron oxide nanoparticles). The haemolytic and cell viability studies show that the biocompatibility of the BSA-iron oxide nanoparticles is improved when compared to the hydrophilic iron oxide nanoparticles without BSA. The SAR value of BSA-iron oxide nanoparticles is also significantly enhanced, irrespective of the size of the iron oxide nanoparticle and method of BSA conjugation.

Our results show that BSA-conjugated FIONs show better biocompatibility and heating characteristics when compared to the BSA-conjugated SPIONs and hydrophilic SPIONs/FIONs without BSA conjugation. It is also evident from our results that BSA conjugation over iron oxide nanoparticles through covalent amide bond formation is better than physical adsorption in improving the biocompatibility and SAR value. To the best of our best knowledge, there are no previous attempts to fabricate a single combinatorial system of BSA-FIONs to address the two main challenges in clinical magnetic hyperthermia-biocompatibility and high heating efficiency.

## Materials and Methods

### Synthesis of Iron Oxide Nanoparticles

The iron oxide nanoparticles were chemically synthesized by the thermal decomposition method as described elsewhere [45, 46]. The chemical composition and physical parameters were modified as per the requirements. Briefly, iron(III) acetylacetonate (Sigma Aldrich) was added to oleic acid (Sigma Aldrich) and benzyl ether (Sigma Aldrich) in required composition and heated under nitrogen purging to 110 °C in order to remove moisture. The temperature was later increased to 160 °C, to initiate nucleation. The reaction was maintained at 280 °C with reflux to promote growth of the iron oxide nanoparticles. The size of the iron oxide nanoparticles depends on the duration of the reaction which is maintained at 280 °C.

We have synthesized SPIONs of size 10 nm and FIONs of size 30 nm. The as-synthesized iron oxide nanoparticles were characterized for their shape and size using Transmission Electron Microscope (TEM, JEOL 100CX). The magnetization properties of the two different sizes—10 nm SPIONs and 30 nm FIONs—were investigated using Vibrating Sample Magnetometer (VSM, Lake Shore Model 7407) and Superconducting Quantum Interference Device (SQUID, QuantumDesign). The size, crystallinity, and purity of the sample were studied using X-ray diffractometer (XRD, Bruker D8 Advanced Diffractometer System with Cu *K*α (1.5418 Å) source).

### Surface Modification of Iron Oxide Nanoparticles

#### Conversion to Hydrophilic Phase

The as-synthesized iron oxide nanoparticles were hydrophobic in nature due to the oleic acid capping. The iron oxide nanoparticles have to be converted to hydrophilic phase so as to use it for in vitro and in vivo applications.

##### Cationic Capping Using CTAB

The strategy of phase transfer using additional hydrophilic molecular layer over the original ligand (oleic acid) of iron oxide nanoparticles was used in this approach. CTAB is a quarternary salt whose hydrocarbon chains adsorb onto the oleic moiety of the iron oxide nanoparticles, allowing the cationic ammonium moiety to face out into the solution, making the iron oxide nanoparticles hydrophilic [47].

Briefly, 0.1 M of CTAB was added to 10 mg iron oxide nanoparticles. The mixture was vortexed and heated to 80 °C. The reaction was stopped after 3 h, and the mixture was washed thrice with ethanol and distilled water. The product was well dispersed and stored in de-ionised water.

##### Ligand Exchange by APTMS

In the ligand exchange strategy, the hydrophilic ligand with more affinity towards the inorganic iron core replaces the original hydrophobic ligand capping the iron oxide nanoparticles, thus rendering hydrophilicity [48].

In our experiments, the silane group of APTMS was exchanged for oleic moiety on the iron oxide nanoparticles. Briefly, 1 mg of iron oxide nanoparticles was dispersed in 10 mL toluene. To the dispersion, 90 µL of APTMS was added and vortexed thoroughly. The mixture was kept in the shaker at room temperature for 72 h for the ligand exchange reaction to take place. The reaction mixture was washed with ethanol and later with distilled water. The product was found to be well dispersed in water.

The hydrodynamic radius and thereby the stability of the hydrophilic iron oxide nanoparticles over a period of time were studied using Dynamic Light Scattering technique (DLS, Malvern Zetasizer Nano-ZS). The coating of surface modified agents over the oleic acid-capped iron oxide nanoparticles was confirmed by Fourier Transform Infra Red spectroscopy (FTIR, Varian 3100).

#### Conjugation of BSA

Conjugation of biomacromolecules like proteins on to the hydrophilic iron oxide nanoparticles, improves the colloidal stability and specificity of the system [49–51]. Generally, the inner side of a protein is hydrophobic while it exposes its hydrophilic amino acid side into the solution. This attribute makes it favourable for researchers to make biocompatible protein-nanoparticle systems [52]. In our experiment, BSA (Sigma Aldrich) was conjugated over the hydrophilic iron oxide nanoparticles in order to render improved biocompatibility and blood circulation.

##### Physical Adsorption using CTAB

In this method of physical adsorption, the cationic CTAB-iron oxide nanoparticles were incubated with anionic BSA. BSA is anionic in nature due to the carboxylate moiety in the amino acids. It can be physically adsorbed on to the cationic CTAB-iron oxide nanoparticles by surface charge interaction. 2 mg mL^−1^ of BSA in 1X Phosphate Buffer Saline (PBS) buffer was added to the hydrophilic CTAB-iron oxide nanoparticles and was left in shaker overnight and washed thoroughly with distilled water.

##### Covalent Amide Bond Formation using APTMS

The APTMS-capped iron oxide nanoparticles have exposed amino groups. The carboxylic groups on the BSA can be activated by the EDC method [53]. A strong amide bond was formed between the carboxylic group of BSA and amino group of APTMS-capped iron oxide nanoparticles. Briefly, 26 mM EDC and 10 mM NHS were prepared in MES (2-(*N*-morpholino) ethane sulfonic acid) buffer. 200 µL of EDC/NHS/MES mixture was added to 1 mL BSA (2 mg mL^−1^) to activate the carboxylic group. 2 mL of APTMS-iron oxide nanoparticles was dispersed in 1X PBS (0.1 mg mL^−1^). The pH was maintained around 7.2–7.4. Both the solutions were then mixed and left in the shaker overnight at room temperature. The reaction was terminated and the product was washed.

**Fig. 1:**
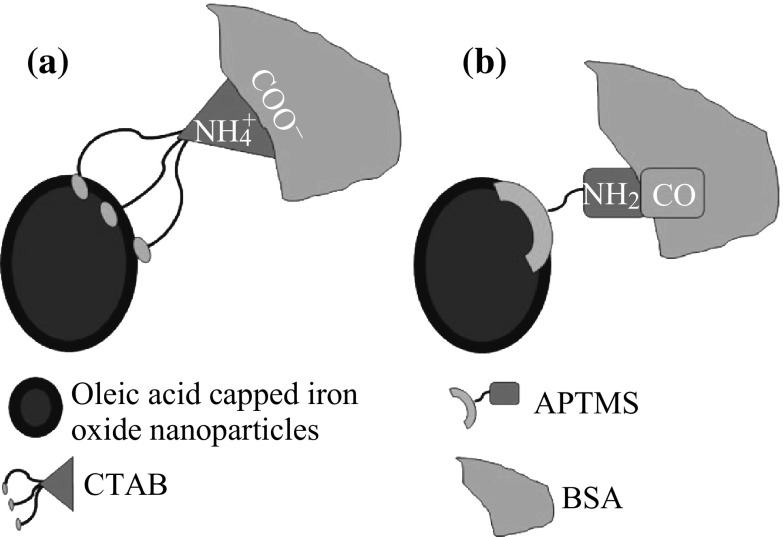
Schematic representation of (**a**) physical adsorption of BSA to CTAB; (**b**) strong covalent amide bond formation between BSA and APTMS

The conjugation of BSA individually over the iron oxide nanoparticles was confirmed using TEM. The hydrodynamic radius and thereby the stability of the BSA-conjugated iron oxide nanoparticles over a period of time were studied using Dynamic Light Scattering technique (DLS, Malvern Zetasizer Nano-ZS). The BSA conjugation over iron oxide nanoparticles was also confirmed using Ultra Violet-Visible Spectroscopy (UV–Vis, Shimadzu UV-1601) and Zetapotential (DLS, Malvern Zetasizer Nano-ZS) studies. Figure 1a, b gives simple schematic representation of the mechanisms behind BSA conjugation using CTAB and APTMS, respectively.

### Improvement of Biocompatibility

#### Blood Aggregation and Haemolytic Studies

Erythrocytes were collected by centrifuging the blood of 5-week-old SCID mice at 700 rpm. The pellet was re-suspended in saline at a ratio of 1:4. The test samples as-synthesized iron oxide nanoparticles, hydrophilic iron oxide nanoparticles, and BSA-conjugated, hydrophilic iron oxide nanoparticles each of ferric ion concentration 0.5 mg mL^−1^ were added to the erythrocytes, and the samples were incubated at 37 °C for 2 h. Distilled water, which leads to 100 % lysis, was used as the positive control as it causes complete haemolysis due to osmosis. 0.1 M NaCl was used as negative control, as it is isotonic with the intracellular solute concentration. Haemolysis of erythrocytes is due to the oxidative stress caused by the test samples on the erythrocytes. The stress ruptures the cell membrane of the erythrocytes and hence the haemoglobin (Hb) leaks out into the solution. The absorbance of the leaked haemoglobin was measured at 540 nm by UV–Vis spectrophotometer (UV–Vis, Shimadzu UV-1601).  % Haemolysis can be calculated using the formula:

$$\% \,{\text{Haemolysis}} = [A_{\rm t} - A_{\rm n} /A_{\rm c} - A_{\rm n} ] \; \times \; 100,$$

where *A*_t_ is the absorbance of the test sample at 540 nm, *A*_n_ is the absorbance of the negative control (0.1 M NaCl) at 540 nm and *A*_c_ is the absorbance of the positive control (distilled water) at 540 nm.

The haemolytic index was also calculated according to ASTM F756-00 standards, according to which, 0–2 % is non-haemolytic; 2–5 % is mildly haemolytic and >5 % is haemolytic [54].

#### Cell Viability Studies

The cell viability assay was performed on healthy Baby Hamster Kidney (BHK) cells (ATCC) in order to compare the biocompatibility of the BSA-conjugated FIONs and hydrophilic FIONs without BSA. Cell Counting Kit-8 (CCK-8) was used to perform the cell viability studies.

Briefly, 100 µL of cell suspension was dispensed into a 96-well plate and pre-incubated at optimum conditions. To the well, 10 µL of BSA-APTMS-FIONs and 10 µL of APTMS-FIONs of final ferric ion concentration of 12.5, 25, 50, and 100 µg mL^−1^ were added. The plate was co-incubated for 24 h. CCK-8 solution of 10 µL was added and further incubated for 4 h. The absorbance at 450 nm was measured using FluoStar Optima microplate reader.

### Enhancement of Heating Efficiency

The magnetic hyperthermia studies to calculate the Specific Absorption Rate (SAR) were carried out by placing the sample inside a copper coil generating an external AC magnetic field. The temperature raise of the sample with respect to the time of exposure of the sample to an AC magnetic field at an amplitude of 32.4 kA m^−1^, frequency of 360 kHz and a magnetic field of 600 Oe was investigated. SAR is expressed as the heat released by the magnetic iron oxide nanoparticles under a magnetic field. The SAR value is calculated from the formula:

$${\text{SAR}} = C_{{{\text{wat}}~}} \times ~\frac{{\Delta T}}{{\Delta t}}~ \times ~\frac{1}{{C_{{{\text{Fe}}}} }}(W_{{\text{g}}} - 1),$$

where *C*_wat_ is the specific heat of the medium (distilled water), 4.18 J (g  °C)^−1^, ∆T/∆t is the the initial slope of the time-dependent temperature curve and *C*_Fe_ is the concentration of ferric ions in the medium, 0.1 mg mL^−1^. The concentration of ferric ions in the samples was determined using ICP-OES analysis (Perkin-Elmer Dual view Optima 5300 DV ICPOES system).

## Results and Discussion

### Characterization of the As-Synthesized Iron Oxide Nanoparticles

**Fig. 2:**
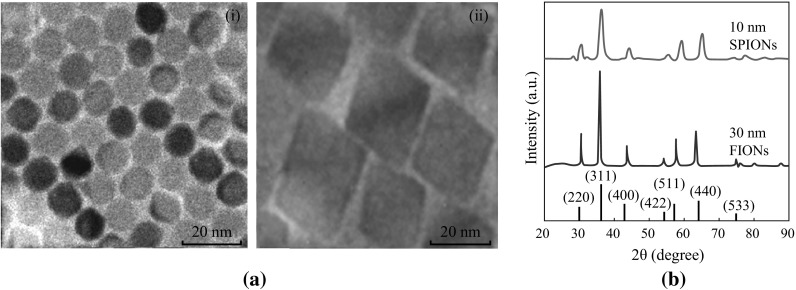
Characterization of as-synthesized iron oxide nanoparticles **a** TEM images of (*i*) 10 nm SPIONs and (*ii*) 30 nm FIONs. **b** XRD plots of SPIONs and FIONs

**Fig. 3:**
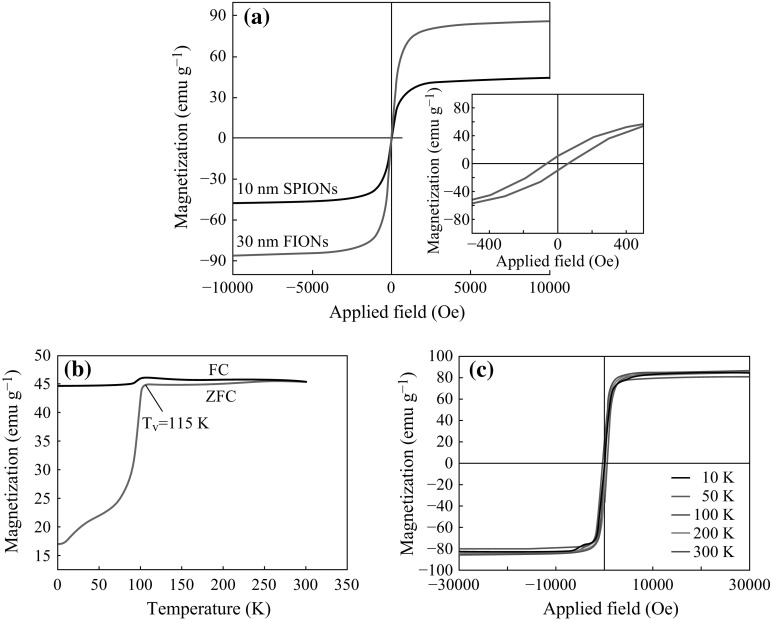
Characterization of magnetic properties of the as-synthesized iron oxide nanoparticles **a** Saturation magnetization values of SPIONs and FIONs using VSM. The *inset* figure shows the coercivity of FIONs. SQUID measurements showing the **b** Verwey transition temperature (*T*_v_) of 115 K deduced from the ZFC plot for FIONs. **c** Magnetization hysteresis loops of FIONs at different temperatures

Two different sizes of iron oxide nanoparticles were synthesized by thermal decomposition method. The size was confirmed by the TEM micrographs from Fig. 2a-i, a-ii. Previous studies have shown that magnetite of 30 nm is ferrimagnetic in nature [55–57]. The XRD plots from Fig. 2b show that the particles are single crystalline and are free of impurities. The size of the iron oxide nanoparticles was also confirmed from the XRD data, using the Scherrer’s formula. The diffraction peaks can be indexed as cubic spinel Fe_3_O_4_ (JCPDS no.19-0629), corresponding to (220), (311), (400), (422), (511), and (440). The peaks became sharper as the particle size was increased. The magnetization properties of the as-synthesized iron oxide nanoparticles are shown in Fig. 3. The saturation magnetization (*M*s) value of the as-synthesized 10 nm iron oxide nanoparticles and 30 nm iron oxide nanoparticles measured by VSM is 45 and 87 emu g^−1^ respectively, as shown in Fig. 3a. The inset in Fig. 3a shows the coercivity exhibited by FIONs. SQUID magnetometer was used to further characterize ferrimagnetic behaviour of FIONs as shown in Fig. 3b, c. The temperature-dependent field cooling (FC) and zero-field cooling (ZFC) magnetization were measured for the FIONs. The Verwey transition temperature (*T*_v_) is the reflection point of the characteristic magnetization jump and is deduced from a ZFC plot derivative as shown in Fig. 3b. The *T*_v_ of the as-synthesized 30 nm FIONs is around 115 K, which is very close to the characteristic *T*_v_ of 120 K for magnetite as reported extensively [58, 59]. The minor shift is commonly observed in magnetite (Fe_3_O_4_) nanoparticles. The magnetization hysteresis loops at different temperatures are shown in Fig. 3c. As observed from the loops, the coercitivity increases as the temperature decreases. All our magnetization studies show the characteristic ferrimagnetic behaviour of 30 nm FIONs. The higher magnetic saturation of FIONs makes it preferred candidate for magnetic hyperthermia application.

### Surface Modification of Iron Oxide Nanoparticles

#### Conversion to Hydrophilic Phase

**Fig. 4:**
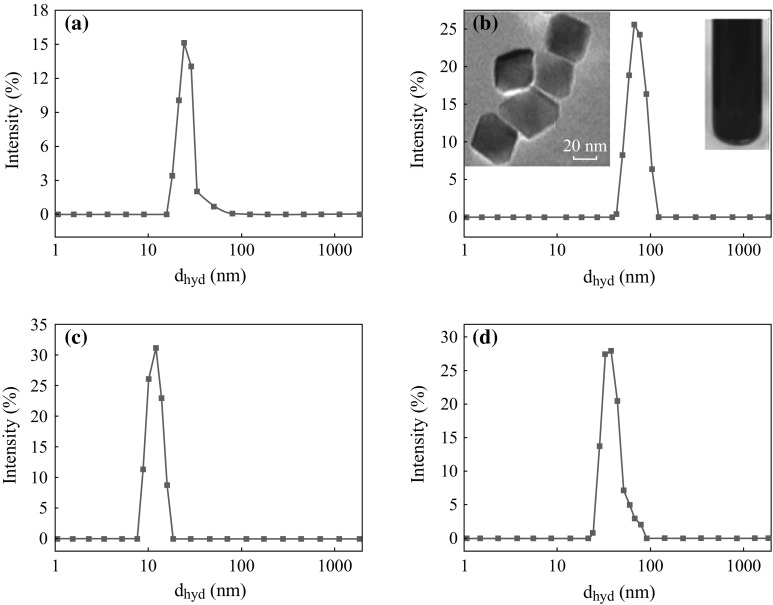
Average hydrodynamic radius of surface modified iron oxide nanoparticles **a** CTAB- SPIONs; **b** CTAB-FIONs; **c** APTMS-SPIONs; **d** APTMS-FIONs. *Inset* images in **b** show the stable solution of CTAB-FIONs and TEM images of the same

**Fig. 5:**
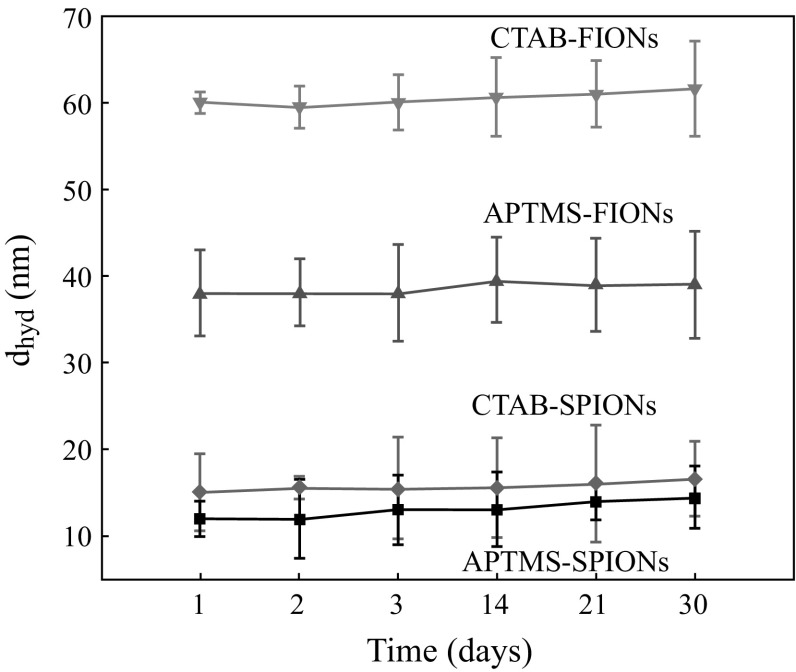
Average hydrodynamic radius of surface modified iron oxide nanoparticles as a function of time

Figure 4a, b shows the average hydrodynamic radius of the CTAB-SPIONs to be 20 nm and CTAB-FIONs to be 60 nm, respectively. The average hydrodynamic radius of APTMS-SPIONs is 15 nm and APTMS-FIONs is 50 nm as shown in Fig. 4c, d, respectively. It was observed from the inset images in Fig. 4b that CTAB forms a very thin additional layer over the FIONs and CTAB-FIONs are stable in the aqueous phase. It is also evident from the DLS graphs that APTMS-coated iron oxide nanoparticles are smaller in size than CTAB-coated iron oxide nanoparticles, as APTMS forms a thin layer of ligand exchange around the iron oxide nanoparticles. Figure 5 shows the hydrodynamic radius of the surface modified iron oxide nanoparticles as a function of time. The iron oxide nanoparticles were quite stable over a period of 30 days. The average hydrodynamic size of surfaced modified SPIONs and FIONs is found to be 17.5 ± 3.5 nm and 55 ± 4 nm, respectively.

**Fig. 6:**
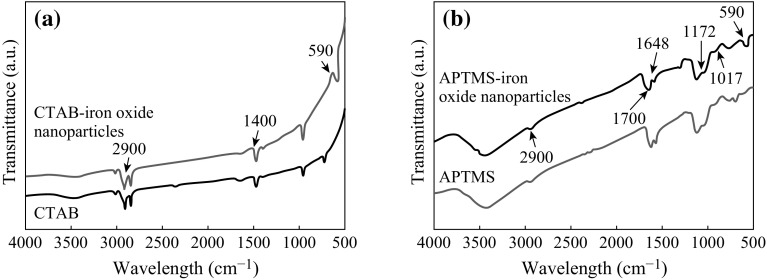
FTIR plots for iron oxide nanoparticles surface modified with (**a**) CTAB (**b**) APTMS

The FTIR peak corresponding to 592 cm^−1^ in both the FTIR plots is related to the Fe–O group and thus confirms the existence of Fe_3_O_4_. The FTIR plot in Fig. 6a confirms that the CTAB was coated over the iron oxide nanoparticles. The peaks corresponding to 2900 cm^−1^ is due to C–H stretching and 1440 cm^−1^ is due to the scissoring vibration of methylene and asymmetric bending mode of the head [N(CH_3_)_3_] methyl group [60].

For the APTMS-coated iron oxide nanoparticles, the peaks at 1700 and 1648 cm^−1^ are due to the stretching vibration of C = O and stretching vibration of C = C bonds, respectively. The peaks at 1172 and 1017 cm^−1^ are due to the stretching vibration of C–O. The asymmetric stretching vibration and scissoring bending vibration of CH_2_ group are represented by peaks at 2926 and 1460 cm^−1^, respectively [61]. This is evident from Fig. 6b.

#### Conjugation of BSA

**Fig. 7:**
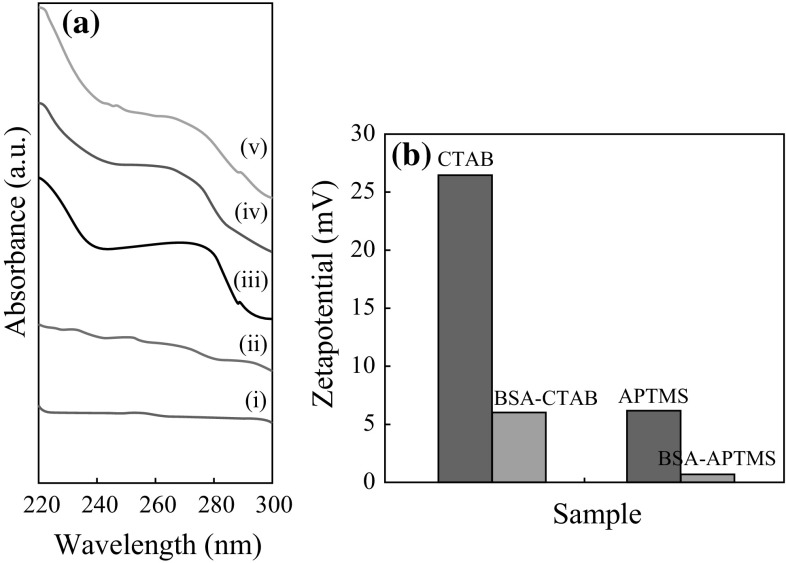
**a** UV–Vis spectrum for (*i*) CTAB-iron oxide nanoparticles, (*ii*) APTMS-iron oxide nanoparticles, (*iii*) BSA (*iv*) BSA-CTAB-iron oxide nanoparticles, (*v*) BSA-APTMS-iron oxide nanoparticles. **b** Zetapotential values surface modified and BSA-conjugated iron oxide nanoparticles

It is also evident from Fig. 7a-i, a-ii that there is no peak observed in the surface modified (hydrophilic) iron oxide nanoparticles, viz, CTAB-iron oxide nanoparticles and APTMS-iron oxide nanoparticles. The characteristic peak of BSA is evident at 280 nm from Fig. 6a-iii. The blue-shift in the UV–Vis spectrum shown in Fig. 7a-iv,a-v proves that the BSA is conjugated to the hydrophilic iron oxide nanoparticles and not merely present in the solution. The disturbances in the polypeptide environment within the BSA due to conjugation of the same to the surface modified iron oxide nanoparticles system are the reason behind the blue-shift. Figure 7b shows the zeta potential comparison of the BSA-conjugated samples and non-conjugated samples. The reduction in surface charge after conjugating with BSA proves that BSA was conjugated with the surface modified iron oxide nanoparticles, as the anionic BSA reduces the zeta potential of the system.

**Fig. 8:**
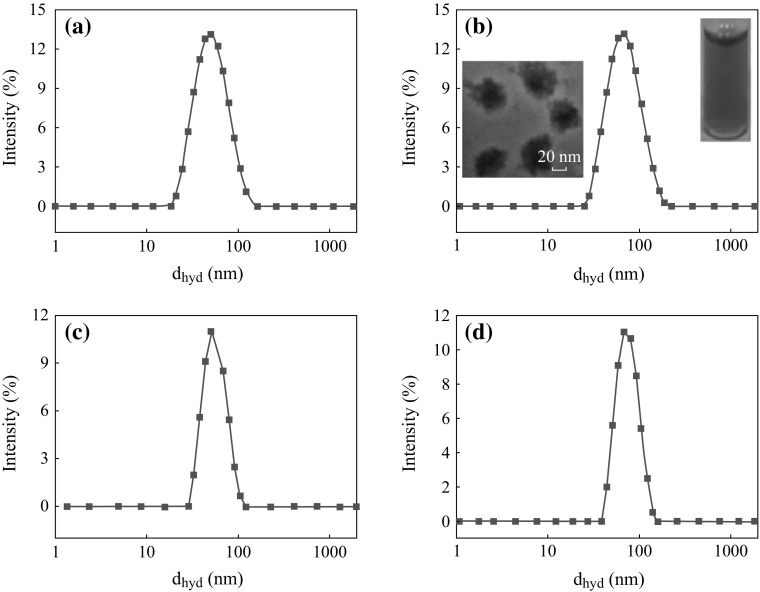
Average hydrodynamic size of BSA-conjugated, surface modified iron oxide nanoparticles **a** BSA-CTAB-SPIONs, **b** BSA-CTAB-FIONs, **c** BSA-APTMS-SPIONs and **d** BSA- APTMS-FIONs. The *inset* images in **b** show the stable colloidal solution and TEM image of BSA-CTAB-FIONs

**Fig. 9:**
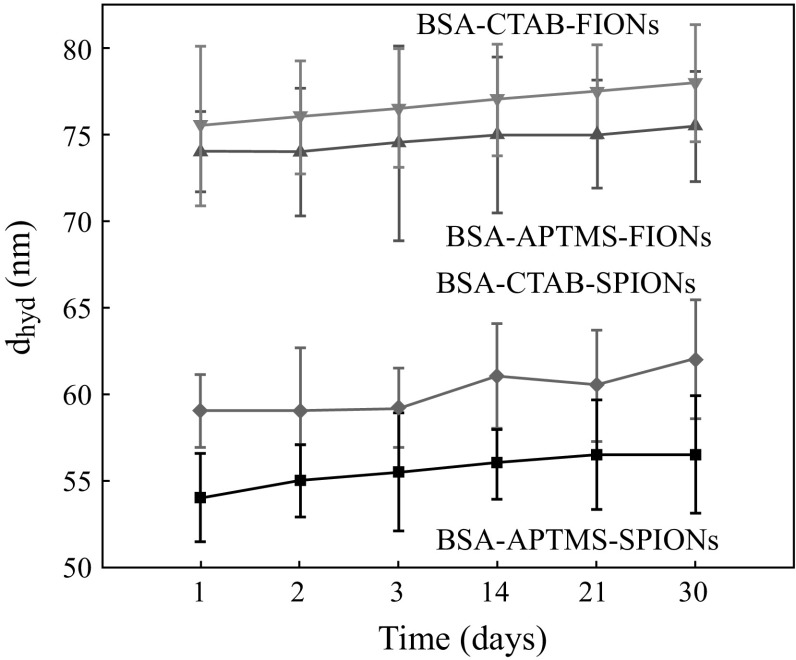
Average hydrodynamic radius of BSA-conjugated, surface modified iron oxide nanoparticles as a function of time

Figure 8a, b shows the average hydrodynamic radius of BSA-CTAB-SPIONs to be 40 nm and BSA-CTAB-FIONs to be 80 nm, respectively. Figure 8c, d shows the average hydrodynamic radius of BSA-APTMS-SPIONs is 30 nm and BSA-APTMS-FIONs is 70 nm, respectively. From the inset images of Fig. 8b, it is evident that the FIONs are individually conjugated by the BSA and are well separated so as to prevent aggregation. The stability of the BSA-conjugated iron oxide nanoparticles was enhanced when compared to the hydrophilic iron oxide nanoparticles without BSA conjugation, irrespective of the method of conjugation. This is evident from the DLS plot over a period of 1 month in Fig. 9. Thus, the average hydrodynamic radius of BSA-SPIONs and BSA-FIONs is around 35 ± 4 and 75 ± 4 nm, respectively. The size range is narrower and is more stable than albumin-conjugated SPIONS reported in previous studies [37, 62].

### Improvement of Biocompatibility

#### Blood Aggregation and Haemolytic Studies

**Fig. 10:**
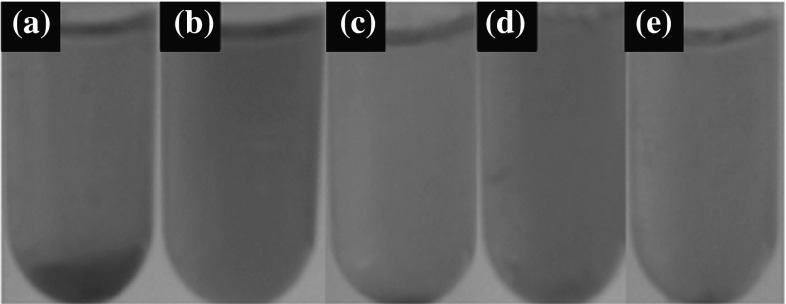
Haemolytic effect of test samples **a** as-synthesized iron oxide nanoparticles; **b** CTAB-iron oxide nanoparticles; **c** BSA-CTAB-iron oxide nanoparticles; **d** APTMS-iron oxide nanoparticles; **e** BSA-APTMS-iron oxide nanoparticles

The photo in Fig. 10a–e shows the effect of test samples on erythrocytes. Figure 10a shows aggregation of ruptured erythrocytes due to as-synthesized iron oxide nanoparticles-SPIONs and FIONs. Due to the high oxidative stress, haemoglobin was released into the solution as the ruptured erythrocytes settled down as debris. Figure 10b, d shows the haemolysis due to hydrophilic iron oxide nanoparticles- CTAB-iron oxide nanoparticles and APTMS-iron oxide nanoparticles, respectively. Mild haemolysis was observed in this case. Figure 10c, e shows that there was a significant reduction in haemolysis after conjugating with BSA. There was almost no cell debris and therefore no haemoglobin leakage in the BSA-conjugated test samples, which implies that cell lysis was almost nil in the same. This is the reason for the reduced absorbance at 540 nm.

**Fig. 11:**
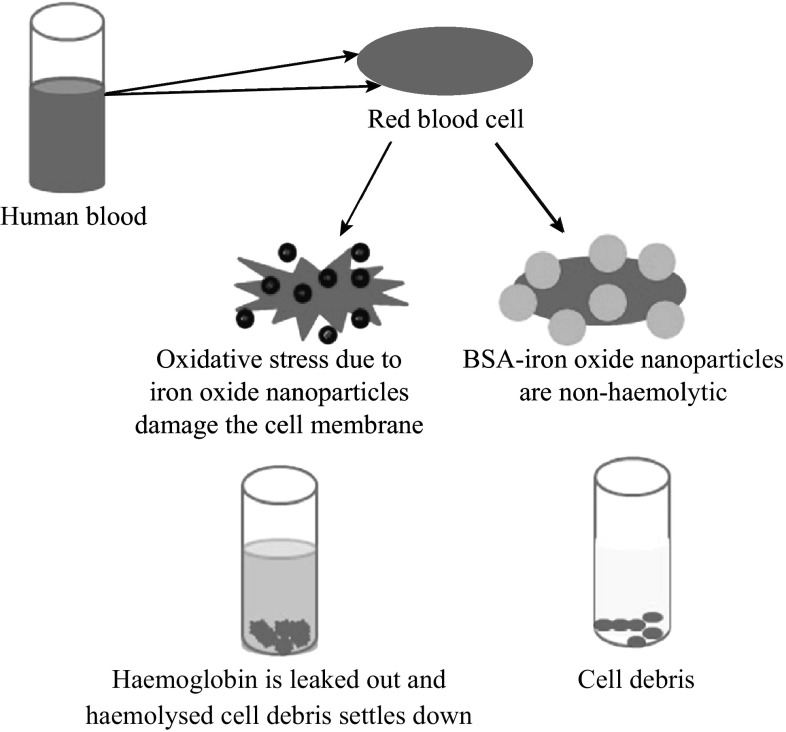
Mechanism of haemolysis

Table 1 shows the UV absorbance values at 540 nm for all the test samples. Figure 11 graphically depicts the general mechanism of haemolysis in both as-synthesized and BSA-conjugated iron oxide nanoparticles.

**Table 1:** Showing the absorbance of haemoglobin at 540 nm

Sample	Absorbance @ 540 nm
Positive control (distilled water)	0.15
Negative control (0.1 M NaCl)	0.002
10 nm SPIONs	0.098
30 nm FIONs	0.12
CTAB-10 nm SPIONs	0.03
BSA-CTAB-10 nm SPIONs	0.0062
CTAB-30 nm FIONs	0.045
BSA-CTAB-30 nm FIONs	0.007
APTMS-10 nm SPIONs	0.04
BSA-APTMS-10 nm SPIONs	0.0083
APTMS-30 nm FIONs	0.058
BSA-APTMS-30 FIONs	0.0093

**Fig. 12:**
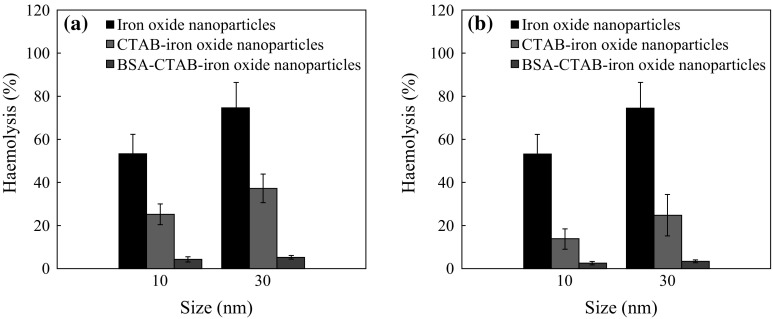
Haemolytic index of the **a** BSA-conjugated CTAB-iron oxide nanoparticles; **b** BSA-conjugated APTMS-iron oxide nanoparticles

The haemolytic studies were conducted as per the ASTM F756-00 standards. It is evident from Fig. 12a, b that the haemolytic index of the as-synthesized hydrophobic SPIONs is 57 % and that of FIONs is 78 %, both well above the 5 % standard, showing that they are highly haemolytic in nature. From Fig. 12a, it is evident that the haemolytic index of CTAB-SPIONs is 28 % when compared to 2.5 % of BSA-CTAB-SPIONs and CTAB-FIONs is 38 % when compared to 2.2 % of BSA-CTAB-FIONs. Figure 12b shows that the haemolytic index of APTMS-SPIONs is 17 % and that of BSA-APTMS-SPIONs is 1.2 %. It also shows that the haemolyic index of APTMS-FIONs is 28 % and that of BSA-APTMS-FIONs is 1.3 %. This shows that BSA conjugation makes the iron oxide nanoparticles almost non-haemolytic nature, as the haemolytic index is ≤2 %, irrespective of the method of conjugation. Surface modification by APTMS is non-haemolytic in nature, when compared to CTAB. This may be because the covalent bonding is stronger than physical adsorption and hence the particles are well conjugated with BSA. It is also observed that BSA-FIONs have biocompatibility similar to that of BSA-SPIONs, even though their size is bigger. Better biocompatibility for a bigger size is an added advantage, as FIONs have better magnetic properties to be used in magnetic hyperthermia experiments.

#### Cell Viability Studies

**Fig. 13:**
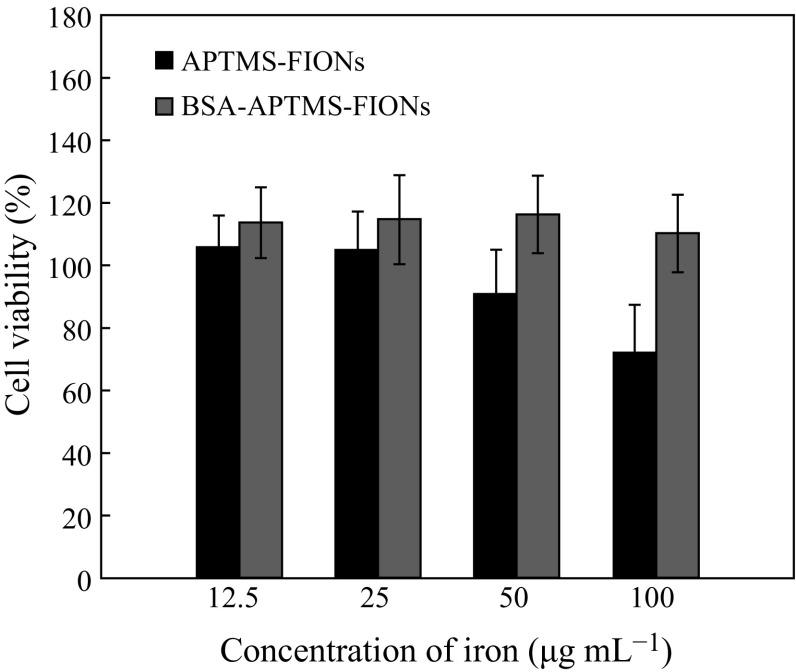
The BSA-conjugated APTMS-FIONs exhibit higher percentage of cell viability than the non-conjugated FIONs

Since APTMS-FIONs and BSA-APTMS-FIONs showed reduced or nil haemolysis, respectively, we performed cell viability studies using the same. The BSA-conjugated APTMS-FIONs show a normalized viability range of up to 120 % (for 12.5 and 25 µg mL^−1^), when compared to the normalized 100 % of APTMS-FIONs (for 12.5 and 25 µg mL^−1^). Previous studies also show that addition of BSA-conjugated SPIONs without the external application of magnetic field show no harmful effect on cell viability [37]. It is also evident from Fig. 13 that the cell viability decreases as the concentration of FIONs in the test sample increases, as in 50 and 100 µg mL^−1^. This shows that BSA-conjugated FIONs exhibit less or no cytotoxicity to healthy BHK cells.

From the haemolytic studies and the cell viability studies, it is evident that BSA conjugation improves the biocompatibility of the iron oxide nanoparticles system. BSA-FIONs show better results when compared BSA-SPIONs or as-synthesized particles or the hydrophilic particles. Thus, the first requisite for an efficient magnetic hyperthermia system is met out.

### Enhancement of Heating Efficiency

**Fig. 14:**
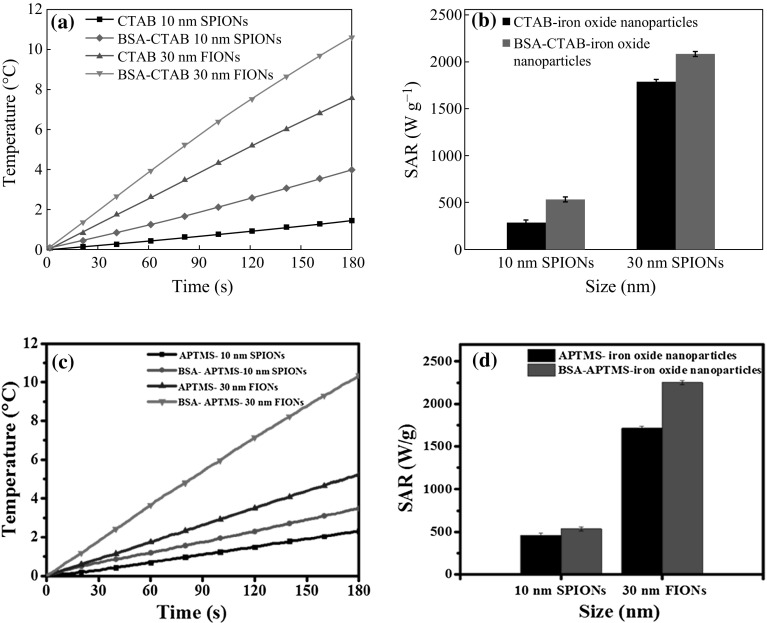
Heating characteristics **a** temperature raise comparison between BSA-CTAB-iron oxide nanoparticles and CTAB-iron oxide nanoparticles; **b** SAR value comparison between BSA-CTAB-iron oxide nanoparticles and CTAB-iron oxide nanoparticles; **c** Temperature raise comparison between BSA-APTMS-iron oxide nanoparticles and APTMS-iron oxide nanoparticles; **d** SAR value comparison between BSA-APTMS-iron oxide nanoparticles and APTMS-iron oxide nanoparticle

The heating efficiency of the hydrophilic iron oxide nanoparticles and BSA-conjugated iron oxide nanoparticles were studied by the magnetic hyperthermia experiments. The SAR value is higher for the BSA-conjugated system, even after the effect of residual BSA was corrected from the system. Figure 14a shows the temperature raise comparison of the CTAB-iron oxide nanoparticles and BSA-CTAB-iron oxide nanoparticles. From Fig. 14b, it is evident that the SAR value of CTAB-SPIONs is 270 W g^−1^ and that of BSA-CTAB-SPIONs is 540 W g^−1^; CTAB-FIONs are 1750 W g^−1^ and that of BSA-CTAB-FIONs is 2200 W g^−1^. Figure 14c shows the temperature raise comparison of APTMS-iron oxide nanoparticles and BSA-APTMS-iron oxide nanoparticles. A temperature raise of around 11 °C is observed in BSA-FIONs, when compared to the 3 °C raise shown by BSA-SPIONs in 3 min. Figure 14d shows that the SAR value of APTMS-SPIONs is 480 W g^−1^ when compared to 520 W g^−1^ of BSA-APTMS-SPIONs; APTMS-FIONs are 1700 W g^−1^ and BSA-APTMS-FIONs are 2300 W g^−1^. It is observed that the covalent bond method using APTMS shows slightly better efficiency irrespective of the size of the particles due to the strong bonding between the hydrophilic iron oxide particles and the BSA and hence improved colloidal stability. It is evident that FIONs of 30 nm size show higher SAR value than SPIONS of 10 nm size, due to their higher saturation magnetization. This makes FIONs better suited candidates for improving the SAR value of the magnetic hyperthermia system, when compared to SPIONs.

The SAR value plots show that, irrespective of the method of conjugation, BSA improves the heating efficiency of the system. While here we report the phenomenon, we are to further investigate the possible reasons behind this interesting phenomenon. It is stated elsewhere that isotropic clusters of BSA are formed under AC magnetic field [63]. Isotropic clusters might have possibly prevented the fibrous aggregation of iron oxide nanoparticles under the AC magnetic field. In general, fibrous aggregation increases the critical size of the nanoparticles in a solution and hence decreases the specific heat of the system. It is well reported that aggregation of nanoparticles decreases the SAR value significantly [40, 64]. Prevention of aggregation by BSA conjugation might have thus enhanced the SAR value of the system. As reported by Samanta et al. in similar studies with SPIONs, this phenomenon could also be attributed to the increased colloidal stability of the BSA-conjugated iron oxide nanoparticles [37]. The BSA-conjugated iron oxide nanoparticles were well separated and well suspended, even under applied magnetic field, whereas the hydrophilic iron oxide nanoparticles without BSA conjugation aggregate in the presence of magnetic field. This interesting phenomenon of improved heating efficiency relating to the colloidal stability imparted by the BSA to the iron oxide nanoparticles will be studied further.

BSA conjugation over FIONs show better SAR value than the BSA-SPIONS, due to the high saturation magnetization. Thus, the second requisite for an efficient magnetic hyperthermia system is also well established.

## Conclusion

We have studied the biocompatibility and heating characteristics of both BSA-SPIONS and BSA-FIONs. The iron oxide nanoparticles were synthesized by the preferred thermal decomposition method using organic solvents. BSA conjugation was done by both physical adsorption and strong covalent amide bond formation.

The haemolytic studies and cell viability studies discussed in the paper confirm that the biocompatibility of the iron oxide nanoparticles increased after BSA conjugation. Particularly BSA-FIONs show better biocompatibility than that of BSA-SPIONs. The improved SAR value of the BSA-conjugated iron oxide nanoparticles system is due to the enhanced colloidal stability and prevention of aggregation. Though the study of efficiency of the surface modifying agents used is beyond the scope of this paper, we still report that BSA conjugation by covalent bonding using APTMS has better colloidal stability and hence better biocompatibility and heating efficiency, as BSA-APTMS-FIONs show better results than that of BSA-CTAB-FIONs. Also the higher magnetic saturation of FIONs leads to higher SAR value and hence better heating efficiency than SPIONs.

We thus conclude that the two key challenges of a very good magnetic hyperthermia system-improved biocompatibility and heating enhancement were addressed through the fabrication of BSA-FIONs. To the best of our knowledge, there are no previous systematic studies on the same. A multivariate therapeutic strategy combining magnetic hyperthermia and chemotherapy using targeted, ligand-conjugated FIONs, could be an extension of our work.
